# Alternative Brain Slice-on-a-Chip for Organotypic Culture and Effective Fluorescence Injection Testing

**DOI:** 10.3390/ijms23052549

**Published:** 2022-02-25

**Authors:** Pedro Herreros, Silvia Tapia-González, Laura Sánchez-Olivares, María Fe Laguna Heras, Miguel Holgado

**Affiliations:** 1 Group of Optics, Photonics and Biophotonics (GOFB), Center for Biomedical Technology, Universidad Politécnica de Madrid, 28223 Pozuelo de Alarcon, Spain; pedro.herreros@ctb.upm.es (P.H.); laura.sanchezo@estudiante.uam.es (L.S.-O.); mariafe.laguna@upm.es (M.F.L.H.); 2 Group of Organ and Tissue on-a-Chip and In-Vitro Detection, Health Research Institute of the Hospital Clínico San Carlos, 28040 Madrid, Spain; 3 Departamento de Neurobiología Funcional y de Sistemas, Instituto Cajal, CSIC, 28002 Madrid, Spain; silvia.tapia@ctb.upm.es; 4 Laboratorio Cajal de Circuitos Corticales (CTB), Universidad Politécnica de Madrid, 28223 Madrid, Spain; 5 Centro de Investigación Biomédica en Red sobre Enfermedades Neurodegenerativas (CIBERNED), ISCIII, 28031 Madrid, Spain; 6 Department of Applied Physics and Materials Engineering, Escuela Técnica Superior de Ingenieros Industriales, Universidad Politécnica de Madrid, 28006 Madrid, Spain

**Keywords:** microfluidics, organ-on-a-chip, brain slice, fluorescence imaging, cell labelling

## Abstract

Mouse brain slices are one of the most common models to study brain development and functioning, increasing the number of study models that integrate microfluidic systems for hippocampal slice cultures. This report presents an alternative brain slice-on-a-chip, integrating an injection system inside the chip to dispense a fluorescent dye for long-term monitoring. Hippocampal slices have been cultured inside these chips, observing fluorescence signals from living cells, maintaining the cytoarchitecture of the slices. Having fluorescence images of biological samples inside the chip demonstrates the effectiveness of the staining process using the injection method avoiding leaks or biological contamination. The technology developed in this study presents a significant improvement in the local administration of reagents within a brain slice-on-a-chip system, which could be a suitable option for organotypic cultures in a microfluidic chip acting as a highly effective bioreactor.

## 1. Introduction

Brain slices preparation under in vitro conditions are a powerful approach that allows neuroscientists to have accurate control over experimental conditions and study neural networks, individual cells, processes, and synapses. For neuroscience research, several experimental 2D and 3D models have been used to study the complexity of brain functions. Although a large number of studies analyze networks of neurons cultured in vitro, 2D cultures cannot simulate brain complexity due to the isolation and lack of contact with other cells [1]. An emerging alternative for the recreation of complex interactions is the culture of human pluripotent stem cells for organoid development. However, high costs and cellular complexity prevent these 3D cultures from being postulated as viable study models [2]. Organotypic brain slice cultures have been widely used for their ability to preserve cytoarchitecture under ex vivo conditions [3], having a very relevant role in studies that have allowed us to know more in-depth processes, such as neurogenesis [4,5], synaptic transmission [6,7], protein expression [8], or responses to physical trauma [9].

There are different approaches for organotypic culture of brain slices. A first approach for mouse hippocampal culture is creating a roll tube culturing system, where the tissue is periodically exposed to air and culture medium [10,11]. A second approach to avoid these problems is interface culturing using porous membranes, where the brain slice is placed on a membrane insert in a multiwell plate, thus the tissue can simultaneously be exposed to culture medium and oxygen [12,13]. However, brain slice cultures have been a great challenge so far, mainly due to the metabolic disintegration that the tissue undergoes due to the limited supply of culture medium and oxygen, waste accumulation, and poor control over the culture environment [14]. Because of this, there was a need to develop new culture methods for brain slice studies. 

In recent years, microfluidics and microfabrication have become powerful tools for tissue engineering, allowing the recreation of miniaturized cell microenvironments with high control and precision [15,16]. The combination of both sciences has led to the concept of organ-on-a-chip, a culture model capable of reproducing tissue equivalents or miniaturized-scale organs [17]. Organ-on-a-chip systems have advantages over roll tube and interface culturing systems: the volumes of culture medium required are lower, prevent the accumulation of depleted media, improve handling culture without risk of contamination, and enable higher control over cell environment. Apart from that, most organ-on-a-chip applications are conducted under flow conditions, leading to real-time screening of secreted molecules [18]. All the benefits of microfluidic systems have been applied to brain slice cultures. The use of continuous flow for brain slice cultures has made it possible to consider an alternative method: submerged culturing, in which the tissue is entirely covered by hyperoxygenated medium [19,20]. This type of culture has better preservation of morphology, but oxygen exchange is less efficient than interface culturing [21,22]. Interphase culturing has been adapted to fluidic systems equally, converging the advantages of the interface culturing systems and microfluidic approaches [23].

Previous works based on fluidic devices for brain slice culture are focused on controlling brain slice microenvironment. There are two different approaches to accomplish this: microperfusion systems [24,25] and microfluidic devices, such as bioreactors [23,26]. This ability to regulate the microenvironment makes microfluidic systems handy tools in the study and development of drugs. Therefore, organ-on-a-chips have been developed to culture brain slices specifically for this bio-application [27,28]. These alternatives arise from certain limitations posed by most fluidic platforms. Specific reagents, such as pharmacological agents or fluorescent dyes, can be dissolved in the reservoir fluid, either by not reaching their optimal concentration or by constantly exposing brain slices to these stimulations. There are brain-on-a-chip devices [29,30,31] focused on localized drug application for brain-on-a-chip, but the alternatives for this application in brain slice-on-a-chip cultures are minimal [28,32]. For this reason, it is necessary to develop alternative methodologies for local delivery in fluidic chips for brain slice evaluation without depending on a flow.

This scientific report presents a new alternative of brain slice-on-a-chip for organotypic culture, integrating an injection system inside the chip to dispense a fluorescent dye. This indicator is used to analyze the slice’s status by fluorescence imaging. The chip presents two interconnected chambers by a permeable membrane and an air bubble pre-chamber in the injection area. This work demonstrates that the injection methodology works correctly and is reproducible through the fluorescence images obtained from the brain slices cultured on the chips.

## 2. Results

### 2.1. Chip Development and Injection System Implementation

**Figure 1:**
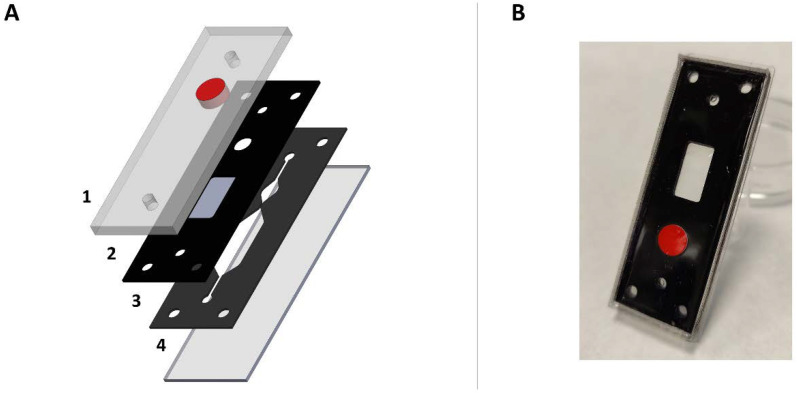
(**A**) 3D exploded view representation of the brain slice-on-a-chip, showing the microfluidic chamber the four layers that shape the chip: 1—polydimethylsiloxane (PDMS) block with two holes for inserting the tubing of the fluidic system, and with an integrated PTFE plug to allow needle puncture of the chip and maintain the chip’s tightness; 2—upper vinyl layer with polycarbonate membrane adhered to the underside; 3—lower vinyl layer through which the flow is perfused. Two compartments form this layout: a prechamber where the reagents are injected, acting as an air bubble trap, and the chamber which interacts with the tissue; 4—75 × 25 mm glass substrate. 2 and 3 present four holes in the corners for a correct overlapping between the vinyl sheets. (**B**) Picture of the final chip version employed for hippocampal slices culture.

The developed engineered chip had two differentiated microfluidic chambers: a lower chamber with flow inlet and outlet, a constant renewal of the culture medium, and an upper chamber that was not subjected to flow (Figure 1). A permeable polycarbonate (PC) membrane delimits both compartments. This chip was subjected to a flow range between 0.1 and 250 µL/min without presenting leaks, keeping the upper chamber airtight, without the perfused flow filling the chamber volume. Additionally, the lower layout featured a pre-chamber to allow reagent injection to avoid bubbles in the chambers. In order to preserve the tightness of the chip, the injection was performed through a polytetrafluoroethylene (PTFE) plug, avoiding potential leaks. The reagent administered through the injection system was a 50 µL of Oregon Green Bapta (OGB) solution (20 µM).

At the preliminary design stage, it was defined that OGB solution must be injected through the lower chamber. There were several reasons to make that decision: it allows a pre-chamber to inject solutions and act as a bubble-trap, maintain the PC membrane’s integrity, and after injection, OGB solution would be mixed with the perfused culture medium. Due to this, the flow could remove the fluorescence solution overload and decrease the background signal.

Initial tests were performed by directly puncturing the upper PDMS block using G25 gauge needles, observing two events: the microfluidic chamber’s cross-contamination and leaks through the PDMS layer. It was also noteworthy that the injection of microvolumes (50 µL) tended to produce bubbles within the fluidic chamber, negatively impacting the laminar flow and microscope observation.

**Figure 2:**
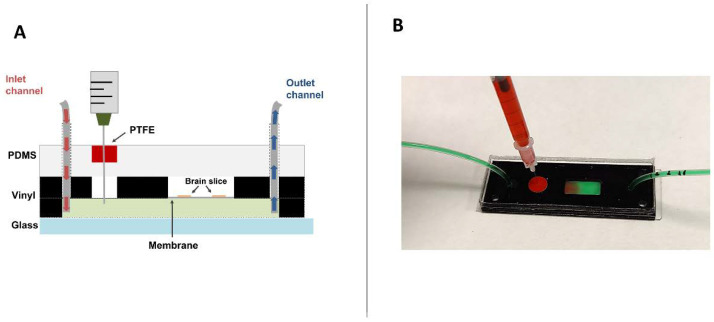
(**A**) Schematic diagram flow of the brain slice-on-a-chip. Hippocampal brain-slices rest over the PC membrane, whereas the culture medium and the OGB solution are perfused across the lower compartment. (**B**) A real injection test replicates Figure 2A. Green fluid is perfused in the lower chamber, and the red fluid is injected through the PTFE plug in the pre-chamber.

After these persistent problems, the final chip included a PTFE plug integrated into the PDMS block and pre-chamber in the lower microfluidic channel (Figure 2). The pre-chamber acted as a bubble trap for air bubbles both present in the flow and those generated by injection. The PTFE plug was capable of self-sealing after being perforated by a needle, preserving the parallel flow inside the microfluidic chamber. During the development stage, the chips were punctured up to five perforations through the PTFE plug, showing no leaks or cross-contamination in any of the chips.

### 2.2. Labeling of OGB in Hippocampal Sections

In order to check the efficiency of the brain slice-on-a-chip device and a staining method by injection system, four chips were manufactured to host two hippocampal slices per chip. An OGB labeling was performed to analyze possible effects along time in viability and integrity of mouse hippocampal tissue inside the chip. 

**Figure 3:**
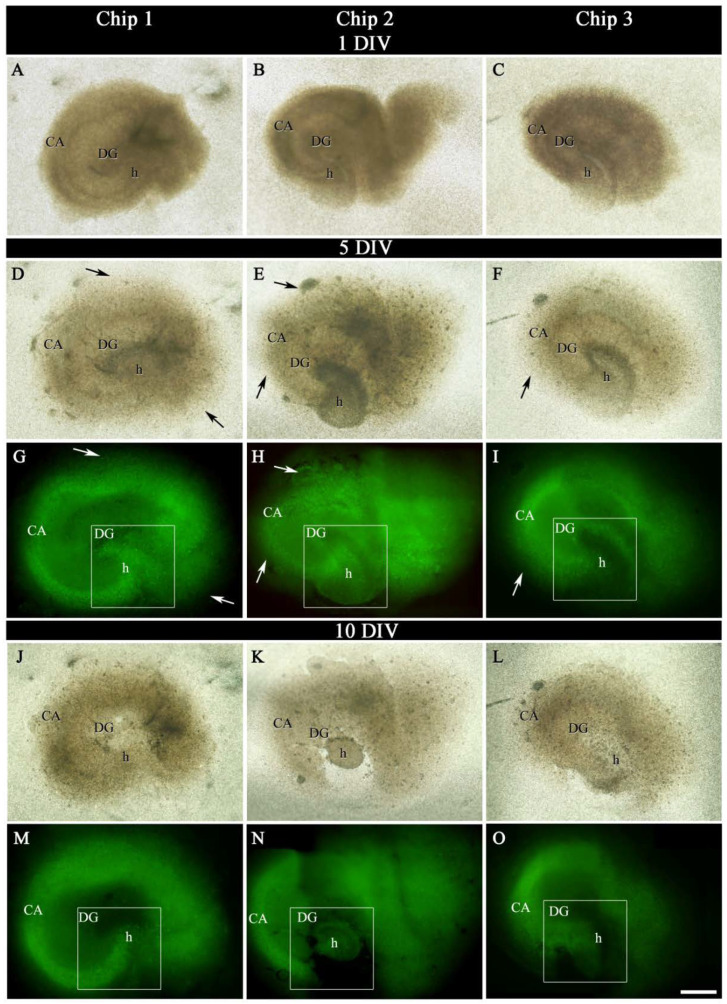
Septal sections of P7 mice hippocampal formation into brain slice-on-a-chip. Low-power photographs from slices placed into Chip 1 (**A**,**D**,**G**,**J**,**M**), Chip 2 (**B**,**E**,**H**,**K**,**N**), and Chip 3 (**C**,**F**,**I**,**L**,**O**) at 1, 5, and 10 DIV. (**A**–**F**,**J**–**L**) showed a progressive change in transparency of the tissue and shrinkage in section thickness from 1 DIV (**A**–**C**) to 5 DIV (**D**–**F**), that was more evident at 10 DIV (**J**–**L**). (**G**–**I**,**M**–**O**), low-magnification photomicrographs demonstrated similar distribution and intensity of OGB marker in the hippocampus section at 5 DIV (**G**–**I**) and 10 DIV (**M**–**O**) in all chips. The OGB marker showed two types of labeling (see also Figure 4) in chip sections along time: a dense dot-shaped labeling (early-medium phase) (**G**,**I**), and a sharp decrease labeling with partial or total lack of OGB labeled cells (late phase) (**M**–**O**) in some zones of the hippocampal subfields. (**D**–**I**) presented a better preservation of the hippocampal cytoarchitecture at 5 DIV compared to 10 DIV. Note, that in (**N**) there was a dramatic reduction of DG size. Solid-arrows display outgrowth of cells from the edge chip section (**D**,**G**). The area indicated by a rectangle in (**G**–**I**,**M**–**O**) was shown at a higher magnification in Figure 3. CA, cornu Ammonis subfield; DG, dentate gyrus; DIV, days in vitro; h, hilus. Scale bar shown in O indicates 500 µm in (**A**–**F**,**J**–**L**) and 400 µm in (**G**–**I**,**M**–**O**).

As shown in Figure 3, OGB fluorescent dye penetrated the tissue, demonstrating the membrane’s permeability and effectiveness of the injection system. 

**Figure 4:**
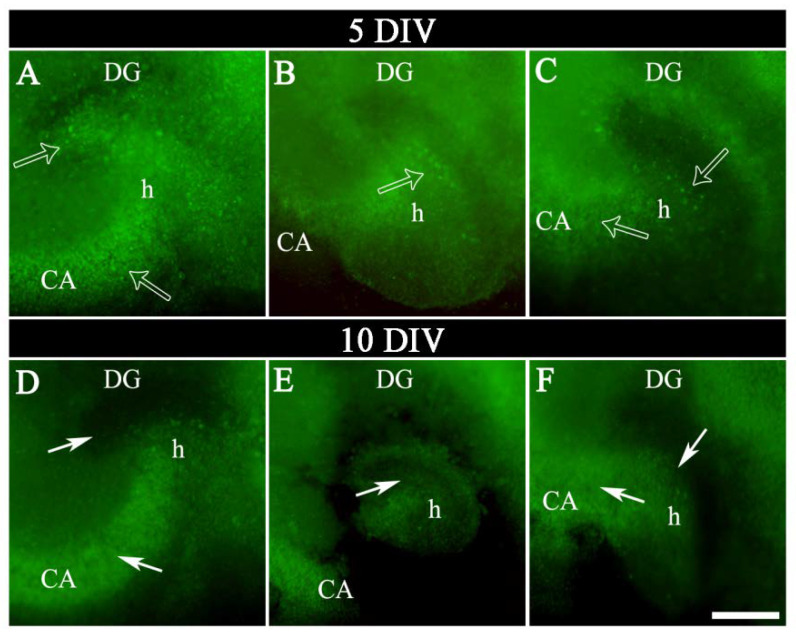
Photomicrographs of hippocampal slices from Chips 1, 2, and 3 illustrating in greater detail the different patterns of OGB marker in CA, DG, and h, at 5 DIV (**A**–**C**) and 10 DIV (**D**–**F**). Note the evident changes in the OGB labeling distribution between 5 and 10 DIV. A dense dot-shaped labeling, corresponding living cells (open arrows) was scattered through whole hippocampal formation, at 5 DIV (medium phase) (**A**–**C**). By contrast, at 10 DIV there was a sharp decrease labeling with partial or total lack of OGB labeled cells (white solid arrows) in some zones of CA, DG, and h (late phase) (**D**–**F**). CA, cornu Ammonis subfield; DG, dentate gyrus; DIV, days in vitro; h, hilus. Scale bar shown in F indicates 270 µm in (**A**–**F**).

**Figure 5:**
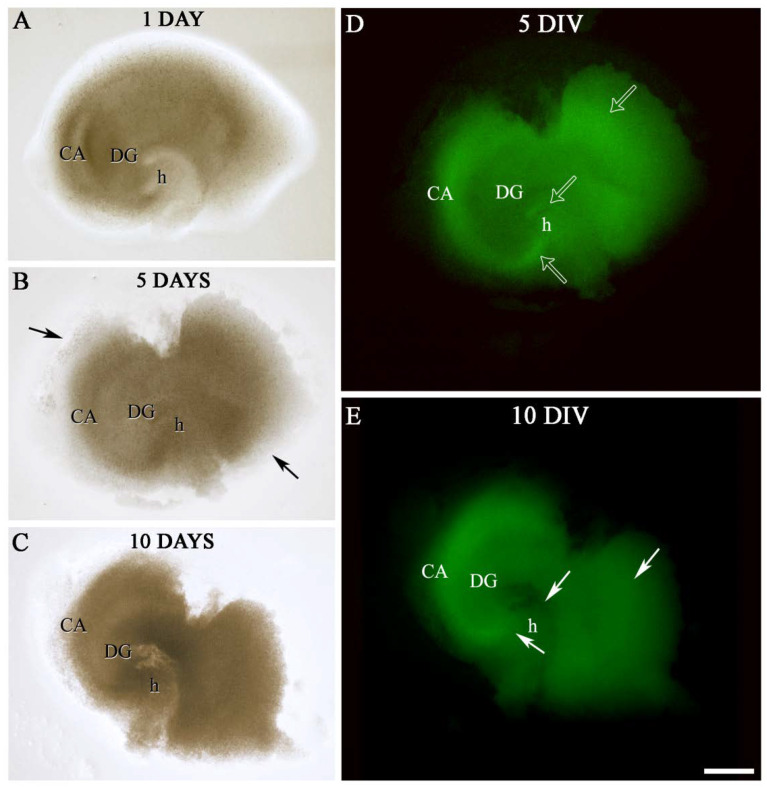
A representative septal section of P7 mice hippocampal formation in OHSC. Low-power photographs from a slice placed onto cell culture insert at 1 (**A**), 5 (**B**,**D**), and 10 DIV (**C**,**E**). (**A**–**C**) displayed a progressive shrinkage of section thickness (black solid arrows) from 1 DIV (**A**) to 10 DIV (**C**) lower than chip slices (**B**) (see also Figure 3). (**D**,**E**) showed the same general pattern of distribution of OGB labeling throughout the hippocampal subregions up to 10 DIV observed in the organ-on-a-chip. Note the changes in the OGB labeling distribution between 5 and 10 DIV. At 5 DIV (medium phase) a dense dot-shaped labeling was observed through whole hippocampal formation (open arrows) (**D**). By contrast, at 10 DIV there was a sharp decrease labeling with partial or total lack of OGB labeled cells (solid arrows) in some zones of hippocampal formation (late phase) (**D**–**E**). CA, cornu Ammonis subfield; DG, dentate gyrus; DIV, days in vitro; h, hilus. Scale bar shown in E indicates 510 µm in (**A**–**C**) and 480 µm in (**D**,**E**).

The viability of the hippocampal sections was observed each day for 10 days in vitro (DIV) in chips and organotypic hippocampal slice culture (OHSC). Since small changes visualized using staining techniques are typically found from measurement to measurement, even using sections from the same hippocampi, subtle changes are difficult to interpret, as previously discussed in several works [33,34]. Thus, the objective of this work was to identify differences between large, obvious differences along time. Therefore, the pattern of fluorescence labeling of OGB was qualitatively analyzed as well as the integrity of the tissue in the chip and OHSC as control (Figure 3, Figure 4 and Figure 5). 

OGB labeled all living cells virtually, which allowed the cytoarchitectonic features of the different hippocampal subregions: cornu Ammonis subfield (CA); dentate gyrus (DG) and hilus (h), and the limits between them to be distinguished, according to the indications of the atlas of the dorsal mouse hippocampus from Bregma −1.46 to −2.30 [35] (Figure 3G–I,M–O and Figure 4). 

Although no quantitative evaluation of living cells number or intensity of fluorescence labeling was performed, differences were found in the pattern of OGB in chip hippocampi sections between 5 DIV and 10 DIV. In general, OGB marker showed two types of staining along time: In 1–5 DIV “early-medium phase”, a dense dot-shaped labeling, in which individual cells were distinguished and scattered through whole hippocampal formation was observed. In 5–10 DIV “late phase”, a worse defined labeling, with partial or total lack of labeled cells in some zones of hippocampal subregions, and an increase of the slice’s background was shown. As shown in Figure 3G–I,M–O and Figure 4, there was a progressive decrease in the labeling and a change in the distribution pattern of the OGB marker mainly in the cornu Ammonis, dentate gyrus, and hilus from 5 DIV, that was more dramatically pronounced at 10 DIV (Figure 3G–I,M–O and Figure 4). 

Besides, a slight and progressive shrinkage, especially section thickness, was detectable at 5 DIV (Figure 3D–F) compared to 1 DIV (Figure 3A–C), which was stronger at 10 DIV (Figure 3J–O). 

Two important criteria for evaluating whether the slices are well-cultured are a change in transparency and cellular spread [1]. As shown the Figure 3, the slices underwent a general change in color and transparency from brownish-opaque at 1 DIV to light brownish-transparent during the first week and outgrowth of cells from the edge chip section. 

No changes in the general pattern of distribution of OGB labeling throughout the hippocampal subregions up to 10 DIV were observed in the OHSC compared to the chip (Figure 5). Furthermore, as shown in Figure 5, the progressive shrinkage of section thickness displayed was smaller than the chip slices (Figure 3). The hippocampal sections were fully attached to the PC membrane at 7 DIV whereas this happens from 14 DIV in the insert membrane, as previously mentioned by other authors [1,36]. However, a better preservation of the typical hippocampal cytoarchitecture in the chip was observed compared to OHSC at 5 DIV (Figure 3D–I and Figure 5). 

## 3. Discussion

The distinctive feature of an organ-on-a-chip system is the generation of a closed continuous system, where any external reagent must be supplemented through the microfluidic set-up. The advantages compared to roll tube culturing and membrane insert cultures are clear: accurate control over the flow and microenvironment, avoiding cross-contamination, and collecting the culture’s metabolic products.

Hippocampal slices are fragile samples susceptible to damage in their cytoarchitecture; disturbances in their maintenance conditions must be avoided in order not to cause mechanical damage to the sample. Due to this, brain slice on-a-chip devices must preserve their hermetic and tightness status to achieve an optimal flow and sterility conditions. These requirements present a disadvantage compared to standard cell culture methodology; traditional multiwell plates have better accessibility to the sample by removing their lids. It is possible to administrate reagents, the most common one being fresh culture medium, but there are more attractive alternatives, such as drugs to analyze the behavior of the sample or fluorescence dyes.

Most current microfluidic systems suitable for mouse hippocampal cultures do not have such accessibility for local tissue interaction because only a single flow is used to preserve the tissue. This limitation generates a series of drawbacks. In the first instance, any potential reactive must be loaded into the inlet reservoir at its optimal concentration. Depending on the reservoir volume, the required amount to reach its optimal dose could significantly increase the study costs. A second drawback would be the exposure time of the stimuli; the biological sample would be subject to overexposure to any reagent found in the input reservoir until it is depleted or changed. Such overexposure could interact harmfully with the culture. Both hindrances are even more critical if the study requires frequent reagent dispensing and could induce a slowdown in the transition to microfluidic models. 

A new reagent injection system was implemented in this brain slice-on-a-chip to overcome the gap between microfluidic and traditional methods, such as roll tube culturing or membrane inserts methods. This methodology allows the local administration into the microfluidic chamber of the chip, interacting instantaneously with the culture avoiding extra microfluidic components to optimize the process. After chip fabrication, it was necessary to test two conditions. On the one hand, if the chip remains watertight, without causing biological contamination and alterations in the internal pressure of the chip after several injections, and on the other hand, if the administered reagent interacts with the sample on the membrane. The fluorescent marker OGB was dispensed through the injection system in every chip to verify those statements. 

The results obtained have demonstrated the methodology’s effectiveness for injecting reagents and the system’s reproducibility. There were no leaks in any of the chips tested through the perforated PTFE plug, and all hippocampi slices were efficiently labeled by fluorescent staining. This second fact confirmed the interaction between the flow of the lower microfluidic chamber and the brain slices through the PC membrane.

Microscope imaging outcomes further highlight better preservation of the typical hippocampal cytoarchitecture in the chip at 5 DIV observed in the present study. This is in line with results obtained by Bakmand T. et al. [23] showing that a fluidic system maintained the characteristic cytoarchitecture seen in hippocampal slices far better than the tissue slices cultured by standard method of organotypic culture. Moreover, the huge presence of OGB living cells [37,38] combined with a change in color to light brownish- transparent of chip sections and outgrowth of cells from the edge chips slices during the first week [1], represent important criteria that show good viability of hippocampal slices inside brain slice-on-a-chips.

The OGB marker enables real-time imaging evaluation of culture viability due to its permeable nature. Nevertheless, additional analysis would provide a more accurate evaluation of the slices. Permanent fluorescence markers can be applied to effectively demonstrate live/dead staining at the finalization of the culture period. In addition, chip material composition allows the extraction of the cultured slices by cutting the PDMS top block and the PC membrane. This feature permits staining protocols where the slice must be fixed as immunostaining, a standard practice for brain slice imaging [39,40]. For future studies, combining OGB real-time analysis and selective labeling of brain cell types by immunostaining as well as live/dead staining would allow a deeper evaluation of brain slice status.

The results obtained from this study prove the effectiveness of this chip for the maintenance of fragile organotypic and allow a local interaction with a fast and straightforward methodology without needing extra components making this chip easy to use.

## 4. Materials and Methods

### 4.1. Design of the Brain Slice-on-a-Chip Device

**Figure 6:**
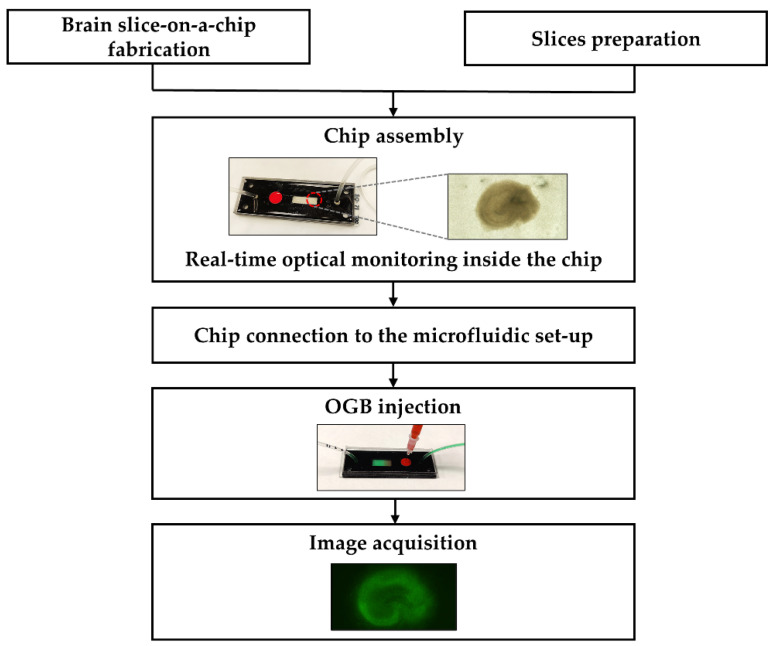
Flowchart showing the main procedures conducted for the brain slice culture and microscope monitoring.

The device was a monolithic chip composed of two interconnected microfluidic chambers delimited by a polycarbonate (PC) membrane (Figure 1). The upper chamber did not present any inlet or outlet connection, and it had a rectangular area exclusively to host the biological sample. The lower chamber presented two different compartments, a pre-chamber where the fluorescent dye was injected and acted as an air bubble trap, and the microfluidic chamber itself, which was in contact with the upper chamber through the PC membrane. The device included a PTFE plug above the pre-chamber to allow the injection of OGB dye inside the chip using a syringe and real-time microscopic monitoring (Figure 6).

### 4.2. Brain Slice-on-a-Chip Fabrication

The materials used in this article and their assembly process are based on a previous work [41], where an organ-on-a-chip composed of glass, vinyl, and PDMS was presented. In this article, these three materials were preserved, and two more components were added—a polycarbonate membrane and a circular PTFE plug (Merck KGaA, Darmstadt, Germany). The dimension of the glass substrate remained at 75 mm × 25 mm, and the vinyl layer was redesigned up to 70 × 25 mm. This area expansion allowed for the introduction of a pre-chamber and a larger main fluidic chamber. Two different vinyl layouts were used for the upper and lower chambers. The lower one was 600 µm wide, and the upper one was 750 µm wide. These chambers were delimited by a polycarbonate membrane (0.4 µm pores, 10 µm wide, Merck KGaA, Darmstadt, Germany). PDMS blocks were made using SYLGARD 184 in a 10:1.5 ratio (elastomer: curing agent) in a mold with the exact dimensions as the vinyl sheets. During the curing process, the PTFE plug was placed in the mold, and subsequently, the elastomer/curing agent mixture was poured into the mold. After the curing process, the plug remained embedded inside the PDMS block, creating a high adhesion between both polymeric materials. Two holes were drilled in the block with a 2 mm puncher to accommodate the microfluidic connections. On the top of the chip, a PDMS block was positioned to seal the device. In order to achieve strong bonding between the PDMS and the upper vinyl layer, the PDMS block had to undergo an oxygen plasma treatment (80 Watts, 120″). This surface activation treatment made possible an optimum vinyl-PDMS bonding [42].

### 4.3. Hippocampal Slices MICE 

Male 7 day-old CD1 mice were obtained from our breeding colony (CTB), maintained under a controlled environment (12-h light/12-h dark cycle; 22 ± 1 °C; ad libitum access to food and water). All the biosafety procedures for handling and sacrificing animals were approved by the bioethics committee from the Research Ethics Committee at the Universidad Politécnica de Madrid and followed the European Commission guidelines for the welfare of experimental animals (2010/63/EU, 86/609/EEC).

### 4.4. Slice Preparation

The hippocampal sections were prepared using a method described by Stoppini et al. [13] with slight modifications. Briefly, P7-CD1 pups were decapitated. The brain was rapidly removed and placed in ice-cold DMEM-high glucose medium. Both hippocampi were dissected under a dissecting microscope and cleaned of the choroid plexus and meninges and transversally cut into 400-µm-thick slices using a tissue chopper VWR. Intact slices from septal hippocampi were then transferred onto the pertinent culture substrate; two hippocampal slices were placed per chip PC membrane ( Merck KGaA, Darmstadt, Germany) and one hippocampal slice per insert membrane of the commercial transwell (Merck KGaA, Darmstadt, Germany).

### 4.5. Procedure of the Brain Slice-on-a-Chip Set-Up

Two hippocampal slices were placed over the membrane in each chip. For a proper chip sealing, the PDMS block underside was previously exposed to a plasma activation, and subsequently, it was attached to the upper vinyl layer. Immediately, the lower microfluidic chamber was filled to have fresh medium using a micropipette to avoid the slice dehumidification, and in addition, inlet and outlet tubings were fitted into the PDMS perforations. Next, a 1 mL syringe (B. Braun, Melsungen, Germany) was charged with cell culture medium DMEM-high glucose, 20% Horse Serum heat-inactivated and 1% antibiotic and antimycotic solution (Thermo Fisher Scientific, Madrid, Spain) and connected to the chip by the inlet tubing. This syringe was positioned into a perfusion pump with a 1 µL/min flow rate being this flow perfused only through the lower microfluidic chamber. A CO_2_ incubator (37 °C, 5% CO_2_) was employed to preserve the microfluidic platform in equal temperature, humidity, and CO_2_ atmosphere conditions for 10 days.

### 4.6. Organotypic Hippocampal Slice Culture

In order to test the effectiveness of the chip, a conventional method of organotypic hippocampal slice culture (OHSC) was used as control for each chip. Briefly, hippocampal sections (400 µm) described above were transferred onto cell culture inserts in P24 multiwell plates and cultivated for 10 days with the same nutrition medium used for the chip and kept in an incubator (37 °C, 5% CO_2_). The depleted medium was changed every third day due to the lack of a continuous flow supply.

### 4.7. Dye Injection and Image Acquisition

The evaluation of brain slice status has been performed by using the fluorescent dye Oregon Green Bapta 488 (06807 Thermo Fisher), a highly sensitive intracellular Ca^2+^ indicator, allowing the acquisition of real-time imaging of living cells. The loading of calcium indicator dye was performed as follows: stock solutions (5 mM) OGB were made using a solution of 20% (*w*/*v*) Pluronic F-127 in absolute dimethylsulphoxide (DMSO). The final volume employed was 50 µL of OGB solution (20 µM) per chip. After 4 DIV, it was injected with a 100 µL Hamilton syringe through the PTFE plug reaching the pre-chamber (Figure 2). An equal volume of OGB was administered for control slices cultured on transwells. Then, sections on the chip, as on the OHSC (control), were incubated for one hour in the culture incubator. After that, the OHSC transwell was transferred to a new well to decrease the fluorescence background signal. Moreover, for a proper penetration of the dye in the chip, a flow of 0.1 µL/min was set for the OGB incubation. In order to elute the excess of OGB, hippocampal slices were visualized one day after (5 DIV). At 9 DIV, this protocol was repeated to obtain images with an optimal fluorescence signal at 10 DIV. During the imaging process, the chip remained sealed, enabling the analysis of the slices inside the chips in real-time. Photographs were acquired with a direct fluorescence microscope Olympus BX51 (Olympus SE & Co. KG, Hamburg, Germany)and inverted fluorescence microscope Leica DMI3000 B (Leica Microsystems, Wetzlar, Germany). 

## 5. Conclusions

A current challenge in culturing mouse brain slices is to preserve the intact cytoarchitecture due to its structural fragility. In this article, a brain slice-on-a-chip device has been fabricated as an alternative culture method for mouse hippocampal slices to improve the culture conditions. This chip allows dispensing microvolumes within the chip independently from the microfluidic system. This attribute allows working with low complexity microfluidic platforms, reducing assay costs. The injection system has been successfully tested, obtaining fluorescence staining in hippocampal slices, proving better preservation of the cytoarchitecture of the brain slices on the chip versus traditional culture methods. Local delivery feature enables the injection of stimuli on the brain slices cultured inside the chip and simultaneously allows the analysis of the tissue’s behavior against these reagents by real-time imaging. The developed methodology opens a new opportunity to apply this organ-on-a-chip alternative for other organotypic cultures with an easy-to-use and disposable device.

## Data Availability

The authors have considered to exclude this statement.

## Abbreviations

DIV	day in vitro
CA	cornu Ammonis subfield
DG	dentate gyrus
DMSO	dimethylsulphoxide
OGB	Oregon Green Bapta
OHSC	organotypic hippocampal slice culture
PDMS	polydimethylsiloxane
PTFE	polytetrafluoroethylene
